# Bioactives in Food-As-Medicine for Special Medical Purposes

**DOI:** 10.1016/j.advnut.2025.100546

**Published:** 2025-10-14

**Authors:** Xueyao Xi, Senyang Hu, Xunuo Zhang, Peng An, Yongting Luo, Junjie Luo, Yinhua Zhu

**Affiliations:** Key Laboratory of Precision Nutrition and Food Quality, Department of Nutrition and Health, China Agricultural University, Beijing, China

**Keywords:** Foods for Special Medical Purposes, Food-As-Medicine, Food-Medicine Homologous, nutritional support, health promotion, disease management

## Abstract

With rising global attention to health and the accelerating trend of population aging, the demand for Foods for Special Medical Purposes (FSMPs) has increased substantially. These products are formulated not only to meet the basic nutritional needs of individuals with specific diseases or physiological conditions but also to provide regulatory physiological effects. In this context, the “Food-As-Medicine” (FAM) concept has gained growing interest. FAM seeks to prevent and treat diseases through the incorporation of functional foods into health management strategies and aligns closely with the traditional Chinese medicine (TCM) theory of “Food-Medicine Homologous” (FMH). Guided by FMH principles, natural bioactive compounds such as polysaccharides, flavonoids, and saponins have drawn significant attention for their anti-inflammatory, antioxidant, and immunomodulatory properties. However, their broader application faces several challenges, including low extraction efficiency, complex purification procedures, and difficulties in ensuring stability, all of which hinder industrial-scale development. This review systematically explores the potential, challenges, and opportunities of FMH-based products in FSMPs, with a particular emphasis on infant nutrition, maternal health, and the management of chronic diseases in the elderly. Our findings highlight the dual value of FMH ingredients in both nutritional support and functional modulation. Furthermore, by integrating FMH theory with modern nutritional science, this review offers a scientific basis for the innovative development of FSMPs. Given the growing global market demand and the increasing dissemination of TCM culture, this field is poised to enter a new phase of development.

**Statement of significance**

This review systematically bridges the traditional theory of Food-Medicine Homology and the development of modern Foods for Special Medical Purposes, focusing on the physiological roles and technical challenges of homologous bioactives in nutrition support for infants, pregnant women, and the elderly, thus offering a theoretical basis and roadmap for advancing precision nutrition in clinical settings.

## Introduction

Amid global population aging and a growing shift toward food-based health strategies, the demand for personalized and precise dietary interventions continues to rise. Foods for Special Medical Purposes (FSMPs) have emerged as essential medical nutrition products designed to meet this need by offering targeted nutritional support. FSMPs are specifically formulated for individuals with defined diseases or physiological conditions, such as impaired food intake, digestive disorders, or metabolic dysfunctions [1]. These products aim to improve nutritional status, support recovery, and reduce hospitalization associated with metabolic or digestive issues [2,3]. Because FSMPs fulfill both nutritional and therapeutic roles, developing innovative ingredients and technologies has become a central focus in the food and health industries.

Alongside rising health awareness, concerns have grown regarding the long-term use of chemical medications, fueling interest in food-based approaches to disease prevention and health maintenance. Within this context, the “Food-As-Medicine” (FAM) concept proposed in Western nutritional science has attracted increasing attention. FAM emphasizes the use of specific foods to prevent and manage both chronic and acute diseases through personalized nutrition strategies [4,5]. A parallel concept, long established in traditional Chinese medicine (TCM), is known as Food-Medicine Homology (FMH), which closely aligns with the principles of FAM. FMH products are defined as “substances possessing both nutritional and medicinal properties, providing nourishment as food while improving health as herbal medicine” [[6], [7], [8], [9]]. Rooted in TCM, FMH was first articulated in the Huangdi Neijing (475–221 BC), which stated, “For the healthy, they serve as food; for the ill, they serve as medicine” [6,[10], [11], [12]]. Globally, concepts like functional foods and nutraceuticals resemble FMH, but key differences remain. Functional foods emphasize isolated bioactives targeting specific effects, whereas FMH relies on the synergistic action of multiple natural components to support overall health. Unlike nutraceuticals, which use purified, standardized extracts in fixed doses, FMH products retain their whole form and act gradually through regular dietary use.

In recent years, incorporating FMH products into FSMPs has shown promising potential, especially for vulnerable groups such as infants, pregnant and lactating women, and the elderly. These products provide essential nutritional support while contributing to chronic disease management, prevention, and improved quality of life. However, despite their broad application potential, several challenges hinder the effective translation of medicinal food-based natural products into FSMPs. These include limitations in ingredient extraction, standardization, and product stability. Addressing these issues is essential to fully harness the benefits of natural FMH products and to develop FSMPs that align with current clinical and nutritional demands.

**FIGURE 1:**
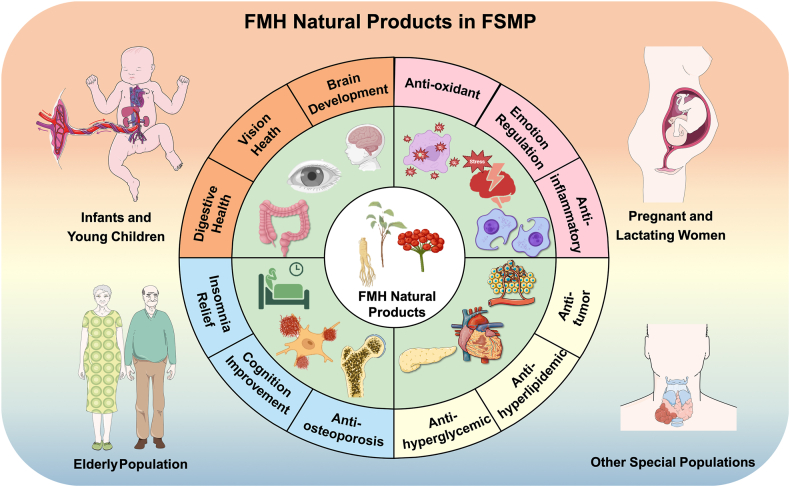
Potential applications of Food-Medicine Homologous (FMH) natural products in Foods for Special Medical Purposes (FSMP) across diverse populations, including pregnant and lactating women, the elderly, and others with unique medical or physiological requirements.

This review explores the potential and current applications of FMH natural products in FSMPs. It highlights their bioactive components, functional roles in different populations (Figure 1), and the challenges and opportunities in their development. The goal is to offer new perspectives for advancing FSMP research and applications using FMH natural products.

## Classification and physiological functions of FMH natural products

**FIGURE 2:**
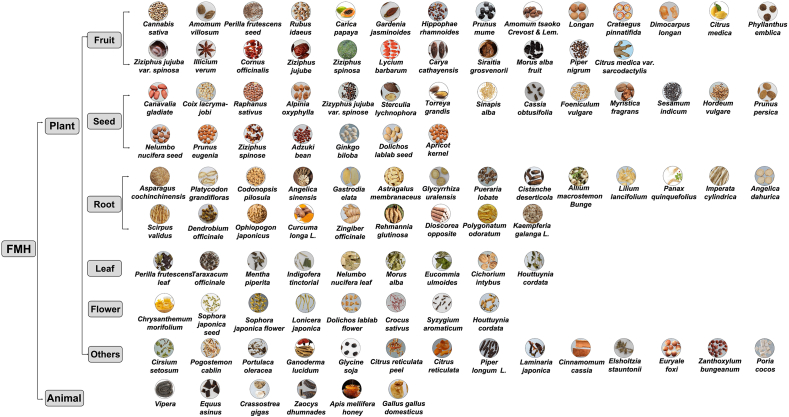
Schematic of Food-Medicine Homologous (FMH) natural products. The diagram displays the 2 main categories of FMH materials: plant-derived (including seed, root, leaf, flower, and others) and animal-derived components.

**FIGURE 3:**
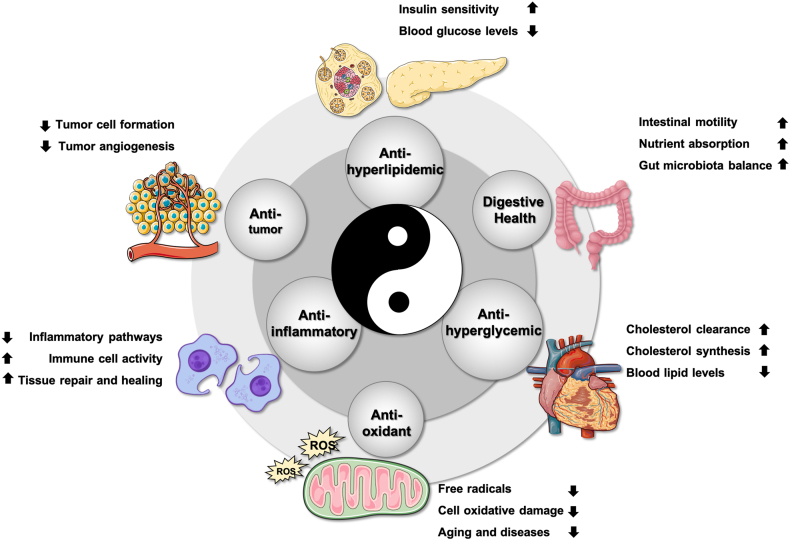
Physiological functions covered by Food-Medicine Homologous (FMH) natural products. This figure illustrates the key physiological functions associated with FMH natural products: antitumor, antioxidant, anti-inflammatory, antihyperlipidemic, and antihyperglycemic effects, as well as digestive health benefits. ROS, reactive oxygen species.

FMH natural products contain diverse bioactive compounds and exhibit multiple pharmacological effects that help prevent cardiovascular diseases, diabetes, and other chronic conditions. Figure 2 illustrates the classification framework of plant-derived and animal-derived FMH natural products, whereas Figure 3 and Table 1 [[13], [14], [15], [16], [17], [18], [19], [20], [21], [22], [23], [24], [25], [26], [27], [28], [29], [30], [31], [32], [33], [34], [35], [36]]summarize the key physiological benefits of FMH natural products, highlighting their strong potential for FSMP development.

**TABLE 1:** Summary of the physiological functions and pathological mechanisms of FMH natural products.

Source	Major active ingredient	Type	Model	Dosage	Effect	Physiological function	Pathological mechanism	Ref.
*Astragalus*	Astragaloside IV	Saponin	CUMS model 6-wk-old male C57BL/6 mice	40, 80 mg/kg/d	↑ Cognitive ability; ↓ TNF-α; ↓ IL-1β; ↓ LPS levels; ↓ Number of microglia in HIP; ↓ Intestinal injury	Anti-inflammatory, improving cognition, Promoting gut health	Astragaloside IV ameliorated CUMS-induced cognitive impairment, inhibited neuroinflammation and restored intestinal barrier damage, but the improvement was mediated by gut microbiota	[13]
*Crataegus pinnatifida*	*Cratae Crataegus pinnatifida spp.* flavonoids	Flavonoid	ICR mice functional dyspepsia model	6 g/kg, once daily, for 30 d	↓ Gastric residue rate; ↑ Intestinal propulsion rate; ↑ Food and water intake; ↑ Abundance of beneficial bacteria and SCFA-producing bacteria; ↓ Abundance of conditionally pathogenic bacteria; ↑ SCFA levels in feces; ↑ Gastrin levels	Relieve dyspepsia	*Crataegus pinnatifida* improves gastrointestinal motility by regulating gut microbiota structure, increasing SCFA production, and influencing neurotransmitters and gastrointestinal hormone levels such as gastrin	[14]
*Citrus reticulata*	*Citrus reticulata* Polyphenol	Polyphenol	Streptozotocin-induced type 2 diabetic male Wistar rats	2.2, 3.4 mL/kg bw/d	↑ Body weight; ↓ Polydipsia, polyphagia, and polyuria; ↓ Fasting blood glucose; ↓ Fat accumulation, and lipid distribution	Blood glucose regulation	—	[15]
*Crataegus pinnatifida*	*Crataegus pinnatifida* polysaccharide (HPS)	Polysaccharide	Human colorectal cancer cell line HCT116	125, 250, 500, 1000 μg/mL	↓ HCT116 cell proliferation; ↑ Apoptosis rate; ↓ Cyclin A1 / D1 / E1; ↑ Caspase-3 expression	Anticancer, induction of apoptosis, inhibition of cell proliferation	HPS regulates cell cycle and induces apoptosis through PI3K/Akt/mTOR and MAPK signaling pathways to exert anticancer effects.	[16]
*Crataegus pinnatifida*	*Crataegus pinnatifida* polyphenols	Polyphenol	Male Wistar type 2 diabetic rats	300 mg/kg, 4 wk	↑ Body weight; ↓ Polydipsia, polyphagia, and polyuria; ↓ Fasting blood glucose; ↓ Fat accumulation, and lipid distribution	Antihyperglycemic, anti-inflammatory, antioxidant, insulin resistance improvement, cardiovascular protection	Through modulation of AMPK/SIRT1/NF-κB signaling pathways, *Crataegus* polyphenols alleviate insulin resistance, inflammation, and improve glucose and lipid metabolism.	[17]
*Camellia sinensis*	*Camellia sinensis* polyphenols	Polyphenol	5-wk-old high-fat-induced lipid metabolism disorder C57BL/6J mice	0.5 mg/mL, 1.0 mg/mL	↓ Body weight; ↓ Serum triglyceride levels; ↓ Inflammatory cytokines (TNF-α, IL-6); ↑ Relative abundance of beneficial bacteria (e.g., Bifidobacteria); ↓ Relative abundance of harmful bacteria (e.g., Enterobacteriaceae) and bacteriophages (e.g., Siphoviridae); ↑ Levels of beneficial metabolites like SCFA; ↓ Levels of harmful metabolites like 3-hydroxybutyrate; ↑ Colonic and ileal mucosal thickness; ↓ Adipocyte hypertrophy	Improve lipid metabolism disorder, anti-inflammatory	*Camellia sinensis* polyphenols improve obesity-related metabolic disorders by regulating gut microbiota and bacteriophage balance, activating the production of beneficial metabolites such as SCFA, and inhibiting the release of inflammatory cytokines (TNF-α and IL-6)	[18]
*Crataegus pinnatifida*	*Crataegus pinnatifida* pectin and hydrolyzed products	Pectin	Male high-cholesterol hamsters	300 mg/kg	↑ Body weight; ↓ Plasma and hepatic total cholesterol; ↑ Fecal bile acid levels; ↓ Atherosclerosis index	Digestive aid, lipid reduction, antilipidemic, Ant-atherosclerosis	*Crataegus pinnatifida* pectin and derivatives modulate cholesterol metabolism by regulating enzymes (e.g., CYP7A1), promote cholesterol conversion to bile acids, and increase bile acid excretion, thus reducing blood lipids and atherosclerosis.	[19]
*Curcuma longa*	Curcumin	Steroid	Ochratoxin A-induced 10-wk-old SD rats	100 mg/kg	↓ Serum ALT, AST, ALP activities; ↑ SOD, CAT, GPx activity; ↓ MDA levels; ↓ Liver inflammation, fatty degeneration, and necrosis	Antioxidant, liver protection	CURC clears free radicals, restores SOD, CAT, and GPx antioxidant enzyme activities, inhibits lipid peroxidation, and alleviates OTA-induced inflammation, improving liver pathological changes.	[20]
*Dioscorea opposita*	*Dioscorea opposita* extract (mainly *Dioscorea opposita* polysaccharides)	Polysaccharide	7-wk-old antibiotic-associated diarrhea model Balb/c mice	4.28, 8.56, 25.68 g/kg·BW·d	↓ Diarrhea rate; ↑ Body weight and cecum index; ↑ Number of probiotics such as Bifidobacteria and Lactobacilli; ↓ Number of potential pathogens such as Enterococcus and Clostridium perfringens; ↑ SCFA levels in feces	Relieve diarrhea, promote gut health	*Dioscorea opposita* improves gut health and alleviates antibiotic-induced diarrhea by promoting the growth of beneficial bacteria, inhibiting the proliferation of harmful bacteria, regulating gut microbiota balance, and increasing SCFA production by providing substrates for SCFA-producing bacteria	[21]
*Gastrodia elata*	Gastrodin	Polyphenol	10-mo-old AD male C57BL/6 mice	50, 100 mg/kg	↑ Spatial learning ability; ↑ Memory ability; ↓ Expression of phosphorylated tau protein, Number of neuronal dendrites; ↓ Neuroinflammatory factors (e.g., IL-1β, IL-18, caspase 1)	Improving cognitive function, sedative hypnotic, antiepileptic effects, neuroprotection	Gastrodin improves learning and memory by reducing neuroinflammation and tau pathology and restoring neuronal and BBB structure and function.	[22]
*Ganoderma lucidum*	Water-insoluble β-(1, 3)-D-glucan	Polysaccharide	LPS-induced RAW 264.7 cells	5, 20, 50, 100 μg/mL	↓ NO and TNF-α production; ↓ iNOS and TNF-α mRNA expression; ↓ Inflammatory response	Anti-inflammatory, Immune regulation	β-(1, 3)-D-glucan inhibits NF-κB and JNK MAPK signaling pathways, downregulating inflammatory genes (*iNOS, TNF-α*) expression to exert anti-inflammatory effects.	[23]
*Hippophae rhamnoides L*	Isorhamnetin, quercetin and kaempferol mixture(SBF)	Flavonoid	PC12 cell line and SH-SY5Y cell line	0, 5, 10, 20 μg/mL and 0, 1, 2, 5, 10 μg/mL separately	↑ Differentiated cells; ↑ Neurofilament expression; ↑ Number of neurites; ↑ PI3K/Akt and ERK1/2 pathways	Promote the development of the nervous system, improving Cognition	*Hippophae rhamnoides L* flavonoids mimic the neurotrophic function of inducing neuronal cell differentiation by activating PI3K/Akt and ERK pathways	[24]
*Lycium barbarum*	*Lycium barbarum* polysaccharide	Polysaccharide	Caenorhabditis elegans	0.1, 0.5, 1.0 mg/mL	↓ Scavenging activity against DPPH radicals, hydroxyl radicals, and superoxide anion radicals; ↑ Lifespan under oxidative stress (0.4 mmol/L H_2_O_2_) and heat stress (35°C) conditions	Antioxidant, antiaging	*Lycium* polysaccharides upregulate daf-16 and its downstream antioxidant genes (e.g., *sod-3, hsp-16.2*) expression to enhance antioxidant capacity and stress response, thereby delaying aging and extending lifespan.	[25]
*Lycium barbarum*	*Lycium barbarum* polysaccharide	Polysaccharide	High-fat fed spotted sea bass	0.75, 1.00, 1.25 g/kg	↑ Weight gain; ↑ Specific growth rate; ↑ Intestinal digestive function; ↑ Intestinal and lipase activities; ↑ SOD antioxidant activity; ↓ ALT, AST, and lipid metabolism biomarkers; ↓ Hepatic fatty vacuolation and balloon-like degeneration	Growth performance improvement, lipid metabolism regulation, antioxidant, liver protection, digestive function regulation	*Lycium* polysaccharides regulate lipid metabolism by downregulating fatty acid synthesis genes and upregulating fatty acid breakdown genes (*PPAR-α, CPT1, ATGL*), enhance antioxidant capacity, reduce oxidative stress, and protect cells from lipid peroxidation damage.	[17]
*Momordica charantia*	*Momordica charantia* polysaccharides	Polysaccharide	High-fat high-sugar diet combined with streptozotocin-induced type 2 diabetic SD rats	150, 300 mg/kg, 4 wk	↓ Fasting blood glucose; ↓ Glucose intolerance and insulin resistance; ↓ Total cholesterol (TC), Triglycerides (TG), and LDL levels; ↑ HDL, SOD, CAT, and GSH levels; ↓ CRP, TNF-α, IL-6 levels	Regulating blood glucose, alleviating insulin resistance, regulating lipid metabolism	By activating AMPK/PI3K signaling pathways, upregulating IRS-1, AKT, and GLUT2 phosphorylation levels, improving insulin resistance and glucose uptake, inhibiting AMPK-mediated hepatic gluconeogenesis, regulating lipid metabolism disorders, and reducing oxidative stress and inflammation.	[26]
*Pueraria lobata*	Puerarin	Flavonoid	Male C57BL/6J mice with high-fat diet-induced obesity	200 mg/kg	↓ Free fatty acids, triglycerides, total cholesterol, and LDL cholesterol; ↑ HDL cholesterol and phospholipids; ↓ Fat tissue macrophage levels; ↓ TNF-α expression	Anti-inflammatory, lipid metabolism regulation	Puerarin reduces M1 macrophages in obese mice, improves blood lipid levels, lowers free fatty acids, triglycerides, and total cholesterol, and improves obesity and its complications through anti-inflammatory and lipid metabolism regulation.	[27]
*Polygonatum*	*Polygonatum sibiricum*	Polysaccharide	Depression model 3-mo-old male C57BL/6 mice	100, 200, 400 mg/kg	↓ Depression-like behavior;↓ IL-1β; ↓ TNF-α; ↑ SOD level; ↓ MDA level; ↓ CORT level; ↑ 5-HT level	Anti-inflammatory, antioxidant, antidepression, regulation of emotion	PSP prevents depression-like behaviors, and synaptic and neuronal damage probably by reducing ROS/HPA axis hyperfunction and the inflammatory response.	[28]
*Poria cocos*	*Poria cocos* polysaccharide	Polysaccharide	Oxidized LDL (ox-LDL)-induced vascular smooth muscle cells	50, 100, 200 μg/mL	↓ Inflammatory cytokines (TNF-α, IL-6); ↓ Oxidative stress markers (ROS, MDA); ↑ Superoxide dismutase (SOD) activity	Anti-inflammatory, antioxidant	PCP activates the ERK/Nrf2/HO-1 signaling pathway and downregulates LOX-1 expression, inhibiting foam cell formation and exerting anti-inflammatory and antioxidant effects.	[29]
*Panax ginseng*	*Panax ginseng* Ginsan polysaccharide	Polysaccharide	Lewis lung cancer model C57BL/6J mice	200 mg/kg	↑ T-cell activity; ↓ Tumor volume; ↑ Gut microbiota diversity; ↑ Mouse survival rate	Immune modulation, antitumor, anti-inflammatory, antioxidant, Gut health protection	Ginsan regulates gut microbiota and its metabolites, increases beneficial bacteria (e.g., Bacteroides, Firmicutes), improves gut health, enhances dendritic cell maturation, and synergizes antitumor immune responses.	[30]
*Portulaca oleracea*	*Portulaca oleracea* phenolic acid	Polyphenol	6–8-wk-old male C57BL/6UC mice	225, 450, 900 mg/kg	↑ Body weight; ↓ Disease activity index; ↓ Serum C-reactive protein and myeloperoxidase levels; ↓ Intestinal inflammation	Anti-inflammatory, gut barrier repair	Repair intestinal barrier function, inhibit NF-κB signaling-mediated inflammation, regulate gut microbiota balance, and improve metabolic disorders.	[31]
			Caco-2 cells	0.1, 0.5, 1, 2, 4, 6 mg/mL	↓ Cell monolayer permeability; ↑ Tight junction proteins (ZO-1, Occludin, Claudin-1) expression; ↓ Inflammatory proteins (iNOS, NLRP3) expression			
*Panax ginseng*	*Panax ginseng* Ginsenoside Re	Polysaccharide	Human colorectal cancer cells (DLD1, HT-29)	10, 50, 100, 200 μM	↓ DLD1 and HT-29 cell proliferation; ↓ Carcinogenic and inflammatory signaling	Anti-inflammatory, antitumor	Ginsenoside Re exerts anticolorectal cancer effects by inhibiting inflammation and tumor-associated signaling pathways.	[32]
		Triterpene saponins	Human-derived colorectal cancer organoids	200 mM	↓ CRC organoid growth			
*Panax ginseng*	*Panax ginseng* Ginsenoside Rb1	Triterpene saponins	C57BL/6J male mice (6–12 wk)	50 mg/kg, intraperitoneal or intravenous injection	↓ Mitochondrial ROS; ↓ Myocardial infarction area; ↓ Cardiac fibrosis; ↑ Cardiac function	Cardiovascular health improvement, antioxidant, antiapoptotic	Ginsenoside Rb1 improves mitochondrial dysfunction, reducing myocardial ischemia/reperfusion injury by inhibiting mitochondrial complex I-mediated ROS production.	[33]
			Primary myocardial cells (H9c2) isolated from adult mice (8–12 wk)	10 μM	↓ Myocardial cell apoptosis; ↑ Cell viability; ↓ Lactate accumulation; ↑ ATP content			
*Panax ginseng*	20(R)-ginsenoside Rg3	Triterpene saponins	Diabetic models Male C57BL/6 J mice (weighing between 18 and 20 g)	10, 20 mg/kg	↓ Body weight; ↓ Blood glucose levels; ↓ Lipid levels; ↓ TG; ↓ LDL-C; ↑ HDL-C; ↓ AGEs; ↑ Insulin; ↓ HbA1c; ↓ VEGFA	Antioxidative damage, protection of vision, anti-diabetes mellitus	Ginsenoside protects the retinal barrier from hyperglycemia-induced damage by activating the Nrf2/HO-1 axis to enhance antioxidant capacity, alleviate ER stress, and reduce apoptosis.	[34]
			HRECs incubated with glucose concentrations (10, 20, 30 and 40 mM) for 24 h	1, 2, 4, 8, 16, 32, 64 μM	↓ Mitochondria ROS level; ↓ HRECs apoptosis			
*Rosa roxburghii*	*Rosa roxburghii* polysaccharides	Polysaccharide	Zebrafish (Danio rerio) tumor xenograft model	100, 200, 400 μg/mL	↓ Tumor growth and metastasis; ↑ ROS and NO generation; ↓ Angiogenesis	Antitumor, Immune modulation	*Rosa roxburghii* polysaccharides activate TLR-2/TLR-4 signaling pathways, modulate immune cell activation, increase ROS and NO generation, and inhibit tumor angiogenesis to reduce tumor growth and metastasis	[35]
*Wolfberry*	Zeaxanthin dipalmitate	Carotenoids	the Pde6brd10 (rd10) C57BL/6J mice, an retinitis pigmentosa model	Light intensity ranged from 230 to 490 lux, 12-h light/dark cycle	↑ Visual ability; ↑ Function of photoreceptors and bipolar cells; ↓ the gene expression related to retinal cells, inflammation, apoptosis and oxidative stress	Anti-inflammatory, antioxidative stress effects	Zeaxanthin dipalmitate by inhibition Multiple pathways play a role, including STAT3, CCL2, and MAPK pathways, while suppressing inflammation in rd10 retinas.	[28]
*Ziziphus jujuba*	Jujube peel polyphenols	Polyphenol	RAW 264.7 cells	1, 5, 10, 50, 100, 200 μg/mL	↑ Cell viability; ↓ TNF-α, IL-1β, IL-6; ↓ ROS and MDA levels	Anti-inflammatory, antioxidant, immune regulation	Jujube peel polyphenols suppress MAPK and NF-κB signaling pathways, activate Nrf2, and reduce inflammation and oxidative stress, exerting anti-inflammatory effects.	[36]

Abbreviations: 5-HT, 5-hydroxytryptamine; AD, Alzheimer’s disease; AGEs, advanced glycation end products; AKT, protein kinase B; ALP, alkaline phosphatase; ALT, alanine transaminase; AMPK, AMP-activated protein kinase; AST, aspartate transaminase; ATGL, adipose triglyceride lipase; BBB, blood-brain barrier; BW, body weight; CAT, catalase; CORT, corticosterone; CRC, colorectal cancer; CRP, C-reactive protein; CUMS, chronic unpredictable mild stress; CYP7A1, cholesterol 7 alpha-hydroxylase; DLD1, human colorectal cancer cell line; DPPH, 2,2-diphenyl-1-picrylhydrazyl; ER, endoplasmic reticulum; ERK, extracellular signal-regulated kinase; FMH, Food-Medicine Homology; GLUT, Glucose Transporter 2; GSH, glutathione; GPx, glutathione peroxidase; HbA1c, Hemoglobin A1c; HIP, Hippocampus; HO-1, heme oxygenase-1; HPA, hypothalamic–pituitary–adrenal; HRECs, human retinal endothelial cells; iNOS, inducible nitric oxide synthase; IRS-1, insulin receptor substrate-1; JNK, c-Jun N-terminal kinase; LDL-C, LDL cholesterol; LOX-1, lectin-like oxidized LDL receptor-1; MDA, malondialdehyde; MAPK, mitogen-activated protein kinase; mTOR, mammalian target of rapamycin; NF-κB, nuclear factor kappa-light-chain-enhancer of activated B cells; NO, nitric oxide; NLRP3, NLR family pyrin domain containing 3; Nrf2, nuclear factor erythroid 2-related factor 2; OTA, ochratoxin A; PCP, *Poria cocos* polysaccharide; PI3K, phosphoinositide 3-kinase; PPAR-α, peroxisome proliferator-activated receptor alpha; PSP, Polygonatum sibiricum polysaccharide; ROS, reactive oxygen species; SCFA, short-chain fatty acids; SOD, superoxide dismutase; SIRT1, sirtuin 1; STAT3, signal transducer and activator of transcription 3; TC, total cholesterol; TG, triglycerides; TLR-2, toll-like receptor 2; TLR-4, toll-like receptor 4; VEGFA, vascular endothelial growth factor A; ZO-1, zonula occludens-1.

↑ significant increase; ↓ significant decrease.

### Classification of FMH natural products

FMH natural products have long played an essential role in traditional diets and medicine by maintaining health and preventing disease. They also offer substantial promise for FSMP development. On the basis of current knowledge, FMH natural products can be classified into 3 main groups: plant-based, animal-based, and others. Plant-based FMH products are the most abundant and include *Syzygium aromaticum*, *Illicium verum*, *Canavalia gladiata*, *Dioscorea opposita*, and *Crataegus pinnatifida*. Animal-based FMH products, though fewer in number, provide important health benefits; examples include propolis and honey. In addition to typical plant and animal sources, certain special types such as *Ganoderma lucidum* and *Laminaria* fall into the “others” category.

In recent years, a growing body of clinical trials and human intervention studies, including randomized controlled trials and systematic reviews, has established a strong scientific foundation supporting the efficacy, safety, and practical application of representative FMH natural products [[37], [38], [39], [40], [41]]. Among these, plant-based FMH products, being the most abundant, are widely used in modern health interventions. A systematic review of global clinical studies on *Panax ginseng* (1979–2018) categorized trials into 5 areas: inflammation, immunity, cancer, glucose metabolism, and cognitive health, highlighting ginseng’s broad therapeutic potential [37]. Notably, a double-blind, multicenter phase II trial involving 66 patients with stage II/III breast cancer demonstrated that *Astragalus* polysaccharides significantly reduced chemotherapy-induced fatigue and improved daily functioning in premenopausal women [38]. Importantly, the large-scale trial demonstrated that a commercial preparation containing astragaloside IV, tanshinone IIA, and ginsenosides (Rb1, Rg1, Re) significantly lowered the risk of cardiovascular death or heart failure hospitalization in 3,119 patients over a median follow-up of 18.2 months [39]. Evidence for animal-based and “other” categories, though less abundant, is also compelling. A systematic review of 20 preclinical and 25 clinical studies found that honey may exert multitargeted antidiabetic effects—antioxidant, hypoglycemic, anti-inflammatory, lipid-regulating, and immunomodulatory, thereby improving glycemic control and reducing oxidative stress-related complications [40]. Similarly, a 90-d randomized, double-blind, placebo-controlled trial in 45 patients with myocardial infarction revealed that polysaccharide peptide extracts from *G. lucidum* may enhance postinfarction recovery through antioxidant and lipid-lowering mechanisms [41]. Collectively, despite disparities in research maturity across FMH categories, the available clinical evidence provides robust support for the utility of these natural products in chronic disease management, subhealth intervention, and nutritional support for special populations.

As of 2025, China’s Ministry of Health has officially listed 106 items classified as both food and medicinal herbs. These items are widely integrated into traditional diets and Chinese medicine practices to regulate physiological functions and prevent diseases. Other regions, including Japan, Europe, and North America, also recognize numerous functional foods, although their definitions and varieties differ [[42], [43], [44]]. The global FMH market is expanding rapidly and is expected to continue growing in the future.

### Physiological functions of FMH natural products

#### Anti-inflammatory effects

Nonsteroidal anti-inflammatory drugs are among the most widely used medications worldwide, but their cardiovascular and renal side effects such as myocardial infarction, hypertension, and kidney dysfunction-have raised increasing concerns [[45], [46], [47]]. These adverse effects have driven researchers to explore natural products, particularly FMH natural products, as potential alternative therapies.

Plant-based natural products like *Portulaca oleracea L.*, *G. lucidum*, and *Poria cocos* play important roles in anti-inflammatory activity. *P. oleracea L.* is a spontaneous herb rich in phenolic acids, flavonoids, alkaloids, and polysaccharides, exhibiting anti-inflammatory, antimicrobial, and antiviral effects [48]. Studies show that *P. oleracea L.* inhibits inflammation-related proteins via the NF-κB signaling pathway, effectively reducing intestinal barrier damage and inflammation in mice, demonstrating potent antiulcerative colitis effects [31]. *G. lucidum* is a medicinal mushroom historically used in Asian countries to treat various diseases and promote longevity [49,50]. Its β-(1,3)-D-glucans inhibit LPS-induced nitric oxide and TNF-α production, downregulate inducible nitric oxide synthase, and suppress NF-κB and c-Jun N-terminal kinase mitogen-activated protein kinase (MAPK) signaling, indicating strong anti-inflammatory properties [23]. *P. cocos*, a well-known medicinal fungus in TCM, contains polysaccharides as its primary active compounds [51]. Research shows that *Poria cocos* polysaccharide (PCP) inhibits inflammatory cytokine expression through the extracellular signal-regulated kinase (ERK)/nuclear factor erythroid 2-related factor 2 (Nrf2)/heme oxygenase-1 signaling pathway and suppresses foam cell formation. Because inflammation in vascular smooth muscle cells plays a crucial role in atherosclerosis pathogenesis, PCP may contribute to treating atherosclerosis through its anti-inflammatory effects [29]. The multifunctional nature of these FMH natural products provides strong scientific support for developing FSMPs with broad-spectrum anti-inflammatory properties.

#### Antioxidant effects

FMH natural products, rich in antioxidant compounds, effectively scavenge free radicals and protect cells from oxidative damage. As a result, they have become a focal point in the development of novel antioxidant therapies. In recent years, *Curcuma longa* has attracted growing interest in Western countries. Curcumin, a polyphenolic compound found in *Curcuma longa* root, acts as a potent antioxidant. It activates antioxidant response elements via the Nrf2 signaling pathway, thereby reducing reactive oxygen species (ROS) and exerting anti-inflammatory and anti-infective effects [52]. A study investigating the protective role of *Curcuma longa* against ochratoxin A (OTA)-induced hepatotoxicity showed that curcumin scavenges free radicals, restores antioxidant enzyme activity, and inhibits lipid peroxidation, significantly mitigating OTA-induced oxidative stress [20]. Similarly, *Camellia sinensis*, a traditional beverage widely consumed in Eastern cultures, is another rich source of natural antioxidants [53]. The polyphenols in *C. sinensis* neutralize free radicals and inhibit the generation of oxidative stress mediators, thereby safeguarding cellular function. Epigallocatechin gallate, a major constituent, not only scavenges free radicals effectively but also stabilizes cell membranes against oxidative injury and enhances endogenous antioxidant enzyme activity [54]. Notably, FMH natural sources such as citrus peel and raspberries are abundant in bioactive compounds like quercetin and resveratrol. These molecules have demonstrated the ability to delay cellular aging and exhibit antioxidant, antitumor, and glycometabolic regulatory effects [[55], [56], [57], [58]].

Oxidative stress plays a critical role in the development of many diseases. Therefore, the informed consumption of FMH natural products not only helps alleviate oxidative stress but also offers valuable insights into the prevention and management of chronic diseases.

#### Antitumor effects

FMH natural products, due to their broad availability, complex phytochemical composition, and low toxicity, show significant promise in antitumor research. Studies have demonstrated that ginsenosides, the major active compounds in *P. ginseng*, such as Ginsenoside Re, exert potent inhibitory effects against various cancers, including colorectal cancer. These compounds induce tumor cell apoptosis, inhibit angiogenesis, and modulate immune responses to suppress tumor progression [32]. In addition, *P. ginseng* polysaccharides enhance immune function, promote apoptosis of tumor cells, and reduce tumor volume, showing marked antitumor activity in lung cancer models [30]. Likewise, *C. pinnatifida* polysaccharides suppress colon cancer cell proliferation by targeting key signaling pathways, including phosphoinositide 3-kinase (PI3K)/protein kinase B (AKT)/mammalian target of rapamycin and MAPK [16]. Polysaccharides from *Rosa roxburghii* activate immune cells to initiate immune responses and simultaneously inhibit angiogenesis, thereby depriving tumors of their nutrient supply and exerting antitumor effects through multiple synergistic mechanisms [35].

The antitumor mechanisms of FMH natural products are complex and multifaceted. These compounds can directly suppress tumor cell growth and metastasis, regulate immune responses, and improve the tumor microenvironment, thereby providing adjunctive therapeutic benefits. A deeper understanding of these mechanisms may facilitate the development of more effective, safer, and diverse drug candidates for cancer treatment.

#### Antihyperglycemic effects

Although traditional oral hypoglycemic agents effectively manage diabetes, their long-term use can cause side effects such as weight fluctuations, gastrointestinal discomfort, skin rashes, and liver damage [59]. As a result, the search for safe and effective natural antihyperglycemic agents has become a critical area of research. FMH natural products, known for their diverse bioactive compounds and favorable safety profiles, show strong potential for lowering blood glucose levels. *Momordica charantia*, a well-established traditional medicinal plant, has been used to treat diabetes since the Ming Dynasty [60]. Its extracts, rich in cucurbitane-type triterpenoids and polysaccharides, improve insulin resistance, enhance insulin sensitivity, and help stabilize blood glucose levels. These effects occur through modulation of disrupted insulin signaling, particularly by activating the PI3K/AKT pathway to enhance glucose uptake and the AMP-activated protein kinase pathway to inhibit gluconeogenesis [26]. *Clausena lansium* (Lour.), studies have shown that it contains essential vitamins, polyphenols, flavonoids, and alkaloids—bioactives known to support metabolic health and prevent chronic diseases [61]. One study reported that polyphenol extracts from *C. lansium* (Lour.) leaves alleviated multiple symptoms in type 2 diabetic rats, including weight loss, excessive sweating, polyphagia, diuresis, hepatomegaly, and hypertrophy of the kidneys and pancreas, while also reducing fasting blood glucose levels [15].

Notably, gut microbiota plays a crucial role in the onset and progression of diabetes. Modulating the gut microbiota balance offers a promising strategy for diabetes management. The multifaceted and synergistic antihyperglycemic mechanisms of FMH products underscore their broad potential for diabetes prevention and adjunctive therapy.

#### Antihyperlipidemic effects

Although conventional antihyperlipidemic drugs can reduce blood lipid levels, they often cause adverse effects such as liver toxicity and gastrointestinal discomfort [62,63]. These limitations underscore the urgent need for safer and more effective alternatives. In recent years, a growing body of evidence has shown that various natural products, particularly those rich in polysaccharides, flavonoids, saponins, and polyphenolic compounds, can effectively regulate lipid metabolism. *Pueraria lobata*, as a commonly used natural agent for lipid lowering, its active compound puerarin has demonstrated multiple beneficial effects. It reduces M1 macrophage populations, lowers levels of free fatty acids, triglycerides, and total cholesterol, and increases HDL cholesterol. These actions collectively alleviate chronic inflammation and improve dyslipidemia [27]. Similarly, polyphenols derived from *C. sinensis* have been shown to modulate the gut microbiota-phage dynamic, enhance the production of beneficial metabolites such as short-chain fatty acids (SCFAs), and suppress the release of proinflammatory cytokines including TNF-α and IL-6. These effects contribute to the attenuation of inflammation and lipid metabolic disorders induced by high-fat diets [18].

FMH natural products not only directly regulate blood lipid levels but also provide adjunctive therapeutic benefits by enhancing lipid metabolism and exerting antioxidant and anti-inflammatory effects. These compounds represent innovative, natural, and safe strategies for preventing and managing hyperlipidemia-related cardiovascular diseases.

#### Digestive health promotion

Gastrointestinal disorders are common but often underrecognized health challenges, especially in vulnerable groups such as patients with cancer, individuals with dysphagia, and those with restricted diets. These populations often suffer from impaired digestion and gut microbiota imbalance, highlighting the urgent need for safe and effective digestive health interventions [64,65]. FMH natural products, known for their strong digestive benefits and high safety profiles, are widely used to manage digestive disorders. *C. pinnatifida*, a widely used medicinal-food herb, has demonstrated efficacy in improving indigestion [66]. In vivo studies further confirm that its extract alleviates functional dyspepsia by modulating gut microbiota, regulating gastrointestinal hormones, and improving motility [14]. *D. opposita*, another well-known traditional remedy for anorexia and diarrhea, demonstrates digestive benefits [67,68]. Supporting this, in vivo studies show its extract alleviates antibiotic-associated diarrhea by modulating microbiota and elevating SCFA production [21].

Improving digestion is essential for nutritional support in patients with cancer and other vulnerable populations. FMH natural products may provide a sustainable source of nutrition, helping these groups better manage disease and treatment-related challenges.

#### Others

In addition to their well-established roles in anti-inflammatory, antioxidant, antitumor, hypoglycemic, lipid-lowering, and digestive functions, FMH natural products also exhibit unique benefits in areas such as vision health, early-life neurodevelopment, cognitive support in the elderly, osteoporosis prevention, emotional regulation, and sleep enhancement.

Regarding visual health, *P. ginseng* Rd and Rb1 improve retinal endothelial cell damage, whereas the rare R-Rg3 shows promise in alleviating diabetic retinopathy by reducing endoplasmic reticulum stress and enhancing retinal function [34,69]. Specifically, a clinical trial confirmed that lutein-rich *Lycium barbarum* improves visual function in early age-related macular degeneration [70].

Regarding neural health, *Hippophae rhamnoides*, rich in flavonoids such as isorhamnetin, quercetin, and kaempferol, mimics neurotrophic effects by activating the PI3K/AKT and ERK signaling pathways, thereby promoting neuronal differentiation [24].

Regarding antioxidant activity, oral administration of *L. barbarum* polysaccharides increased antioxidant enzyme activity and reduced malondialdehyde, a biomarker of oxidative damage in aged mice [71]. Astragaloside IV, a potent polysaccharide from *Astragalus,* significantly improved cognition and behavior in mice exposed to chronic unpredictable mild stress [13].

Regarding bone health, *Astragalus* polysaccharide prevents intracellular and mitochondrial ROS accumulation, restores proliferation of bone marrow mesenchymal stem cells, inhibits apoptosis and senescence, and reverses the downregulation of pluripotency genes *Nanog, Sox2*, and *Oct4* [72].

Regarding emotional health, *Polygonati* polysaccharides exhibit antidepressant-like properties by reducing reactive oxygen species, restoring hypothalamic–pituitary–adrenal axis function, and suppressing inflammation [28]. In addition, *Ziziphi spinosae* semen, widely used in clinical settings for insomnia and anxiety, significantly reduces locomotor activity and anxiety-like behaviors in sleep-deprived zebrafish models [73].

Health concerns such as vision loss, impaired brain development, cognitive decline, osteoporosis, emotional dysregulation, and insomnia are strongly associated with aging and quality of life. Natural products derived from FMH play a vital role in supporting overall health.

## Application of FMH natural products in FSMPs

**FIGURE 4:**
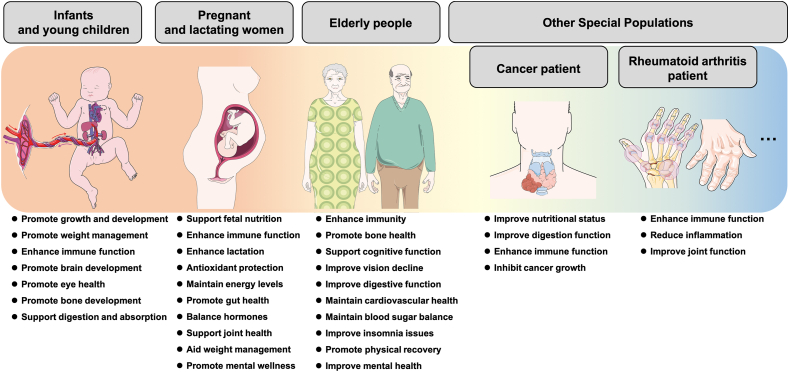
Characteristics of Foods for Special Medical Purposes (FSMP) requirements across different age groups. This figure classifies key nutritional and health needs for: (A) infants and young children, (B) pregnant/lactating women, (C) elderly people, and (D) special populations (including those with cancer, rheumatoid arthritis, etc.). Specific functional benefits are highlighted, including immunity enhancement, bone health support, cognitive function maintenance, and management of metabolic and sleep disorders.

In recent years, the development of FMH-derived natural products as FSMP has shown significant potential, particularly for vulnerable populations such as infants and young children, pregnant and lactating women, and the elderly. Understanding the physiological and metabolic changes, as well as the specific nutritional requirements of these groups, remains critical. Figure 4 illustrates the varying FSMP needs across age groups, emphasizing the importance of tailored nutritional and therapeutic support.

### Global and domestic classification of FSMPs

To better understand how FMH-derived bioactive components apply in FSMPs, it is essential to clarify the regulatory classification systems used in different countries. These systems directly influence the development, approval, and clinical application of FSMPs containing functional ingredients. Globally, FSMP classification typically depends on product function, target population, and nutrient composition.

Represented by major regions such as the European Union, the United States, Japan, and China, although there are differences in specific classification systems and terminology, all are committed to providing precise nutritional support tailored to the physiological and clinical needs of diverse populations. Regulatory frameworks in these regions commonly emphasize that product formulations should closely align with the metabolic characteristics and health status of the target population. For example, the European Union focuses on disease specificity and metabolic needs in its classifications; the United States strictly limits FSMPs to physician-supervised medical foods; Japan establishes detailed categories based on life stages and physiological conditions; and China balances nutritional completeness with disease specificity. Collectively, these regulatory systems provide a solid foundation for the safe, effective, and targeted application of FMH-derived bioactive components in FSMPs. A comparative overview of FSMP classifications and regulatory frameworks in these key regions is presented in Table 2.

**TABLE 2:** Comparison of FSMP classification and regulatory frameworks in China, the Europe, Japan, and the United States.

Region	Main classification of FSMPs	Notes and features	Regulatory authority/document
European Union	Complete Nutritional Formula Foods Specific Disease Formula Foods Foods for Special Metabolic Needs	Designed for patients with functional impairments, including those recovering postoperatively, with specific diseases, or metabolic disorders (e.g., phenylketonuria)	European Commission, Commission Directive 2013/46/EU on foods for special medical purposes, 2013.
United States	Medical Foods (under physician supervision)	Medical foods can make disease-related claims, whereas dietary supplements are limited to general nutritional support and not considered FSMP	The United States Food and Drug Administration, Medical Foods Guidance Documents & Regulatory Information, 2020.
Japan	Infant formulas Foods for pregnant/breastfeeding women Foods for elderly with chewing/swallowing difficulties Medical foods Foods for Specified Health Uses	Highly detailed categorization covering different life stages and physiological needs	Ministry of Health, Labour and Welfare, Japan, Standards for Foods for Special Dietary Uses, 2018.
China	Complete Nutritional Formula Foods Specific Complete Nutritional Formula Foods Non-Complete Nutritional Formula Foods	Complete and specific complete formulas can serve as the sole source of nutrition; noncomplete formulas are intended as partial supplements used alongside other foods	Standardization Administration of the People’s Republic of China, National Food Safety Standards General Principles for Special Medical Purposes Formula Foods, 2013.

Abbreviation: FSMP, Foods for Special Medical Purposes.

Regional differences in FSMP classification directly impact the formulation, labeling, and marketing strategies of FMH-derived bioactives. A clear understanding of these regulatory frameworks is essential for developers to ensure therapeutic efficacy, regulatory compliance, and global scalability of FMH-enriched FSMPs.

### Demand characteristics for FSMPs in special populations

#### Infants and young children

Infancy and early childhood represent a critical period for the growth and development of children and their families [74]. Consequently, implementing evidence-based dietary practices and providing targeted nutritional supplementation are essential for supporting healthy development in this population.

The demand for FSMPs in infants and young children primarily centers on several key aspects. First, growth and weight management are essential, as early malnutrition affects millions and accounts for 45% of child mortality [75,76]. Second, enhancing immune function is vital, as infants are born with immature immunity and high infection risk [77,78]. Third, Nutrition in the first 1000 d is critical for brain development and future mental outcomes [75,79]. Fourth, impaired early visual development may associate with cognitive deficits and persistent visual health problems [80]. Fifth, promoting bone development is fundamental to infant growth, as the infant skeleton undergoes rapid changes [81,82]. Finally, early gut microbiota development is closely linked to infant health, functional ingredients that help establish a healthy gut are essential [83,84].

When developing FSMPs for infants, several considerations are essential. First, carefully control ingredient dosage to avoid excessive potency, especially with components like *L. barbarum* and *Fructus Aurantii*. Second, use appropriate forms such as purees or powders to enhance digestion and absorption. Third, monitor for allergic reactions closely; any adverse symptoms should prompt discontinuation and consultation with healthcare providers. Finally, regularly assess product effectiveness and adjust formulations based on the infant’s growth and developmental progress (Table 3) [[85], [86], [87], [88], [89], [90], [91], [92], [93], [94], [95], [96], [97], [98], [99], [100], [101]].

**TABLE 3:** Overview of the application of FMH natural products in FSMPs for infants.

Demand characteristics	Sources	Main active ingredients	Application effects	Application recommendations	Ref.
Enhancing immune function	*Astragalus membranaceus*	Astragalosides, astragalus polysaccharides	Boosts immunity, reduces infection risk, and supports immune system health in infants	1. Control dosage infants and young children have relatively weaker constitutions, and therefore ingredients with strong bioactivity, such as *Lycium barbarum* and *Ziziphus jujuba*, should not be used in excess. The dosage should be strictly controlled according to the properties of the ingredients. 2. Refine ingredient forms complementary foods for infants should ideally be prepared in a puree or powdered form to facilitate better digestion and absorption. Harder ingredients, such as *Juglans regia* L. and *Ganoderma lucidum*, should be crushed and mixed with other ingredients to enhance safety. 3. Monitor for allergic reactions If symptoms such as rashes, vomiting, or other discomforts appear, the ingredient should be discontinued immediately, and medical consultation should be sought. 4. Regular effect evaluation Regularly track the growth and development of infants, monitoring indicators such as weight, height, and appetite, to assess the effectiveness of special medical foods.	[85]
	*Ganoderma lucidum*	*Ganoderma lucidum* polysaccharides			[86]
	*Lycium* barbarum	*Lycium barbarum* polysaccharides, *Lycium barbarum* flavonoids			[87]
	*Lonicerae japonicae*	Chlorogenic acid, *Lonicerae japonicae* flavonoids			[88]
Promoting bone development	Ostreidae	Ostreidae polysaccharides, amino acids	Supports bone and dental health, providing essential minerals		[89]
Promoting brain development	Fish oil	Fatty acids	Rich in ω-3 fatty acids, promoting memory, learning ability, and overall cognitive function in infants		[90]
Promoting eye health	*Chrysanthemum*	Chrysanthemum flavonoids (e.g., Quercetin)	Promotes eye health, protects against UV damage, and improves night vision		[91]
	*Lycium barbarum*	*Lycium barbarum* polysaccharides, *Lycium barbarum* flavonoids			[92]
	*Morus alba*	Anthocyanins, flavonoids			[93]
Promoting growth and development	*Panax ginseng*	Ginsenoside Rg1, Ginsenoside Rb1	Promotes physical development, enhances physiological function, and supports immune system health		[94]
	*Ziziphus jujuba*	*Ziziphus jujuba* polysaccharides, flavonoids, amino acids			[95]
Promoting weight management	*Cinnamomum cassia*	Cinnamon polysaccharides, cinnamon flavonoids	Helps manage healthy weight in infants and young children, stimulates appetite, and increases nutrient intake		[96]
	*Crataegus pinnatifida*	*Crataegus pinnatifida* flavonoids, *Crataegus pinnatifida* acids			[97]
	*Dimocarpus longan*	*Dimocarpus longan* polysaccharides, amino acids			[98]
Supporting digestion and absorption	*Dioscorea opposita*	*Dioscorea opposita* polysaccharides, triterpenes	Improves digestive function, promotes intestinal health, and relieves bloating and constipation		[99]
	Honey	Sugars, natural enzymes			[100]
	*Poria cocos*	*Poria cocos* polysaccharides, triterpenes			[101]

Abbreviations: FMH, Food-Medicine Homology; FSMP, Foods for Special Medical Purposes; ω-3, omega-3 PUFAs.

#### Pregnant and lactating women

Pregnant and lactating women undergo profound physiological changes that create unique health demands worldwide. The health needs of them span physiological, psychological, and maternal–fetal domains. First, adequate nutrition during pregnancy is critical, as they directly affect maternal health, breast milk quality, and fetal outcomes [102]. Second, pregnancy-induced immunosuppression increases infection risk; immune support remains essential [103,104]. Third, as pregnancy progresses, increased oxidative stress and hormonal fluctuations may contribute to complications such as hyperemesis gravidarum [[105], [106], [107]]. Fourth, hormonal changes also cause indigestion and constipation, affecting ∼32.4% of pregnant women globally [102,108]. Fifth, hormonal shifts during pregnancy may impair metabolism, requiring regulation to prevent gestational diabetes, which affects over 16 million pregnancies annually and increases maternal risk of type 2 diabetes by 22-fold [[109], [110], [111]]. Sixth, significant weight fluctuations during pregnancy and postpartum can stress bones and joints, potentially leading to osteoporosis or fractures, therefore making calcium, vitamin D, and related nutrients vital for skeletal health [112,113].

Beyond physical health, psychological well-being during pregnancy and lactation is equally important. A large-scale study of over 183,000 women found that fatigue peaks in the first trimester, whereas sleep disturbances worsen throughout pregnancy [114]. Similarly, anxiety and related disorders affect ∼20% of pregnant and postpartum women, with certain conditions, such as obsessive-compulsive disorder, demonstrating increased risk during and after pregnancy [115].

Proper formulation and use of FMH natural products are crucial for pregnant and lactating women. Due to common gastrointestinal sensitivity, ingredients such as *D. opposita* and *G. lucidum* should be cooked or powdered to improve absorption and avoid raw or potent forms. Overuse of blood-tonifying or energy-enhancing components (e.g., *Astragalus*, honey) may strain the circulatory system, potentially causing edema or hyperglycemia, so these should be used in moderation. Seasonal adjustments are also important: warming herbs like *Zingiber officinale* and *Cinnamomum cassia* can support circulation in winter, whereas cooling herbs such as *Astragalus* and *Lonicerae japonicae* help reduce internal heat during summer. These choices impact not only maternal health but also breast milk composition, requiring careful ingredient selection and formulation (Table 4) [[85], [86], [87], [88], [89],[94], [95], [96], [97], [98], [99],[116], [117], [118], [119], [120], [121], [122], [123], [124], [125], [126], [127], [128], [129], [130], [131]].

**TABLE 4:** Overview of the application of FMH natural products in FSMPs for pregnant and lactating women.

Demand characteristics	Sources	Main active ingredients	Application effects	Application recommendations	Ref.
Antioxidant protection	*Gardenia jasminoides*	Gardenia glycosides, flavonoids	Reduce oxidative stress, slow the aging process, improve skin health	1. Adjustment of Ingredient Form During pregnancy, gastrointestinal sensitivity, loss of appetite, and difficulty in absorption are common. It is recommended to powder or simmer ingredients like *Dioscorea opposita*, *Lycium barbarum*, *Ganoderma lucidum*, and *Codonopsis pilosula* to facilitate absorption. Avoid consuming raw or overly strong medicinal herbs. 2. Avoid Excessive Supplementation Excessive intake of blood-tonifying or Qi-tonifying ingredients may overload the maternal circulatory system, leading to issues such as edema or elevated blood sugar. Ingredients such as *Astragalus, Angelica,* and honey should be consumed in moderation. 3. Enhance Physical Strength and Relieve Fatigue In the later stages of pregnancy, physical strength tends to decline. Ingredients like *Codonopsis, Dimocarpus longan,* and *Litchi chinensis,* which provide sustained energy, can help alleviate symptoms such as fatigue and low energy. 4. Seasonal adjustments Pregnant women should adjust their choice of medicinal and edible ingredients according to the season. For example, in winter, warming ingredients like *Zingiber officinale* and *Cinnamomum cassia* can be used in moderation to promote blood circulation. In summer, cooling ingredients such as *Astragalus, Lonicerae japonicae* and *Morus alba* should be favored to clear heat and detoxify. Seasonal adjustments may also influence the composition of breast milk during the lactation period.	[116]
	*Morus alba*	Anthocyanins, vitamin C			[117]
	*Vitis vinifera* L seeds	Resveratrol, anthocyanins			[118]
	*Zingiber officinale*	Curcumin			[119]
Bone and joint health	*Astragalus membranaceus*	Astragalosides, *Astragalus* polysaccharides	Strengthen bone density, prevent pregnancy-related osteoporosis, maintain bone health		[120]
	*Ostreidae*	Calcium, selenium, zinc			[89]
Fetal development and nutritional support	*Astragalus*	*Astragalus* polysaccharides, astragalosides	Promote healthy fetal development, support normal development of the nervous system, bones, and immune system		[85]
	*Panax ginseng*	Ginsenosides Rg1, Rb1			[94]
	*Ziziphus jujuba*	*Ziziphus jujuba* polysaccharides, flavonoids			[95]
Immune function enhancement	*Ganoderma lucidum*	*Ganoderma lucidum*polysaccharides, triterpenoids	Strengthen maternal immunity, reduce the risk of infection during pregnancy, protect the fetus from pathogens		[86]
	*Lycium barbarum*	*Lycium barbarum* polysaccharides, goji flavonoids			[87]
	*Lonicerae japonicae*	*Lonicerae japonicae* glycosides, chlorogenic acid			[88]
Maintain endocrine balance	*Astragalus membranaceus*	*Astragalus* polysaccharides, astragalosides	Regulate hormone levels, maintain endocrine balance, support healthy pregnancy		[121]
	*Angelica sinensis*	Angelica flavonoids, angelica polysaccharides			[122]
	*Pueraria lobata*	Puerarin, isoflavones			[123]
Maintain physical strength and relieve fatigue	*Codonopsis pilosula*	Codonopsis polysaccharides, codonopsis flavonoids	Enhance physical strength, reduce pregnancy fatigue, improve energy levels, help maternal recovery		[124]
	*Dimocarpus longan*	*Dimocarpus longan* polysaccharides, amino acids			[125]
	Honey	Glucose, fructose, amino acids			[126]
Promote digestion and gut health	*Dioscorea opposita*	*Dioscorea opposita* polysaccharides, diosgenin	Improve gut health, regulate gastrointestinal function, promote digestion and absorption		[99]
	*Poria cocos*	*Poria cocos* polysaccharides, triterpenoids			[101]
	*Zingiber officinale*	Gingerol, curcumin			[127]
Promote lactation	*Angelica sinensis*	*Angelica* polysaccharides, *Angelica* flavonoids	Stimulate milk production, promote breast milk supply, ensure sufficient nutrition for the infant		[128]
Promote weight management	*Cinnamomum cassia*	Cinnamon polysaccharides, *Cinnamomum cassia* flavonoids	Help control weight gain during pregnancy, prevent excessive weight gain, ensure healthy weight management		[96]
	*Crataegus pinnatifida*	*Crataegus pinnatifida* flavonoids, triterpenoids			[97]
Support mental health and emotional stability	*Glycyrrhiza uralensis*	Glycyrrhizin, licorice polysaccharides	Promote emotional stability, alleviate anxiety, improve sleep, support mental health		[129]
	*Lilium brownii* var. *viridulum*	*Lilium brownii* var. *viridulum* polysaccharides, flavonoids			[130]
	*Poria cocos*	*Poria cocos* polysaccharides, triterpenoids			[131]

Abbreviations: FMH, Food-Medicine Homology; FSMP, Foods for Special Medical Purposes; VC, vitamin C.

#### Elderly population

As the global population ages, age-related diseases increasingly determine both healthy lifespan and overall life expectancy [132,133]. First, aging impairs immune function, increasing susceptibility to infections and making immune support a critical component of FSMP [134,135]. Second, osteoporosis-related fractures have become a major public health concern, significantly affecting quality of life [136]. Third, Alzheimer’s and other dementias dominate age-related cognitive decline, posing a growing health burden [137]. Fourth, visual impairments such as blurred vision and dry eyes are prevalent and may progress to glaucoma or cataracts if left untreated [138]. Fifth, over half of adults over 50 experience digestive issues, impacting overall health [139]. Sixth, age-related sarcopenia further decreases metabolic rate and physical endurance, necessitating targeted interventions to restore energy [140,141]. Beyond organ-specific decline, elderly individuals face a growing burden of chronic diseases. Over 20% of seniors globally have diabetes, projected to surpass 276 million by 2045 [[142], [143], [144]]. Similarly, cardiovascular aging further contributes to life-threatening conditions such as hypertension, which remains a leading global cause of mortality with a prevalence that has continued to rise over the past 3 decades [133,145].

In addition to physical health challenges, psychological well-being in the elderly has received growing attention. First, ∼40% of seniors experience sleep disturbances, which are associated with Alzheimer’s disease and metabolic disorders [141,[146], [147], [148], [149], [150]]. Second, loneliness and depression are linked to chronic disease and reduced quality of life in the elderly [151].

When using FMH, elderly individuals should adhere to specific dietary principles to maximize therapeutic benefits and minimize health risks. First, due to diminished digestive capacity with age, high intakes may lead to indigestion or bloating. Adopting a "small meals, more frequent" strategy, along with suitable preparation methods such as powdering or stewing can improve digestibility. Second, ingredient-drug interactions must be carefully managed. For example, although *C. pinnatifida* and *G. lucidum* aid in lipid regulation, *P. ginseng* may interact with anticoagulants, increasing the risk of bleeding. Therefore, professional supervision and routine monitoring of blood pressure and lipid levels are crucial. Third, dietary diversity is essential. Prolonged use of a single FMH ingredient can lead to nutritional imbalances or adverse effects. Rotating among herbs such as *P. ginseng*, *Astragalus*, and *L. barbarum* supports immune function while reducing dependency on any one component. Lastly, emotional disturbances are prevalent among older adults. FMH ingredients like *Ziziphus jujuba* and *Dimocarpus longan* may alleviate anxiety, enhance sleep quality, and support emotional health (Table 5) [92,99,101,124,127,[129], [130], [131],[152], [153], [154], [155], [156], [157], [158], [159], [160], [161], [162], [163], [164], [165], [166], [167], [168], [169], [170], [171]].

**TABLE 5:** Overview of the application of FMH natural products in FSMPs for elderly population.

Demand characteristics	Sources	Main active ingredients	Application effects	Application recommendations	Ref.
Enhancing immunity	*Astragalus membranaceus*	*Astragalus* polysaccharides, Astragalosides	Enhance immune response, regulate immune system function, improve disease resistance	1. Small, frequent meals Elderly individuals often experience difficulty chewing and reduced digestive function. To avoid indigestion and discomfort, smaller, more frequent meals are recommended. Cooking methods like grinding or stewing can enhance digestibility. 2. Avoiding ingredient interactions Some ingredients, such as *Crataegus pinnatifida* and *Ganoderma lucidum,* have lipid-lowering effects, whereas *Panax ginseng* may interact with anticoagulants, increasing bleeding risk. It is essential to monitor for potential interactions and consult a healthcare professional, regularly checking health indicators like blood pressure and lipid levels. 3. Maintaining a diverse diet Relying on a few medicinal foods can lead to nutritional imbalances. A varied diet ensures comprehensive nutrient intake and reduces the risk of side effects from prolonged use of any single ingredient. Rotating ingredients like *Panax ginseng*, *Astragalus*, and *Lycium barbarum* can help prevent adaptation. 4. Emotional stability and sleep regulation Elderly individuals may face emotional challenges, such as anxiety or mild depression. Medicinal foods like sour jujube seeds, *Dimocarpus longan*, and *Lilium brownii* var. *viridulum* bulbs can help alleviate anxiety and improve sleep quality.	[152]
	*Lycium barbarum*	*Lycium barbarum* polysaccharides, flavonoids			[153]
	*Panax ginseng*	Ginsenosides (Rg1, Re)			[154]
Improving digestive function	*Dioscorea opposita*	*Dioscorea opposita* polysaccharides, Dioscorea saponins	Promote gastrointestinal motility, aid digestion, improve constipation		[99]
	*Poria cocos*	*Poria cocos* polysaccharides, Triterpenoids			[101]
	*Zingiber officinale*	Curcumin, Gingerol			[127]
Improving insomnia	*Dimocarpus longan*	*Dimocarpus longan* polysaccharides, Terpenoids	Improve sleep quality, promote deep sleep, alleviate anxiety, calm the mind		[155]
	*Morus alba*	*Morus alba* polysaccharides			[156]
	*Rosa rugosa* Thunb.	Rose terpenoids			[157]
	*Ziziphus spinosa*	*Ziziphus* saponins, Flavonoids			[158]
Improving mental health and emotional stability	*Glycyrrhiza uralensis*	Glycyrrhizin, Licorice polysaccharides	Relieve anxiety and depression, improve sleep, promote emotional stability		[129]
	*Lilium brownii* var. *viridulum*	*Lilium brownii* var. *viridulum* polysaccharides, flavonoids			[130]
	*Poria cocos*	*Poria cocos* polysaccharides, Triterpenoids			[131]
Improving vision decline	*Chrysanthemum morifolium*	*Chrysanthemum* flavonoids, Essential oils	Improve vision, alleviate night blindness, delay macular degeneration and other eye issues		[159]
	*Lycium barbarum*	*Lycium barbarum* polysaccharides, flavonoids			[92]
Maintaining blood sugar balance	*Astragalus membranaceus*	*Astragalus* polysaccharides, Astragalosides	Lower blood sugar, enhance insulin sensitivity, assist with diabetes management		[160]
	*Momordica charantia*	*Momordica charantia* saponins, polysaccharides			[161]
Maintaining cardiovascular health	*Crataegus pinnatifida*	*Crataegus pinnatifida* flavonoids, acids	Improve blood circulation, reduce blood pressure, improve cardiovascular function		[162]
	*Dimocarpus longan*	*Dimocarpus longan* polysaccharides			[163]
	*Ganoderma lucidum*	*Ganoderma lucidum* polysaccharides			[164]
Promoting bone health	*Crocus sativus*	Crocin	Enhance bone density, prevent osteoporosis, improve bone health		[165]
	*Portulaca oleracea*	Flavonoids (quercetin, rutin, etc.), alkaloids (portulacine)			[166]
Promoting physical recovery and alleviating fatigue	*Astragalus membranaceus*	Astragalosides, *Astragalus* polysaccharides	Enhance physical strength, alleviate fatigue, improve physical recovery		[167]
	*Codonopsis pilosula*	*Codonopsis* polysaccharides, flavonoids			[124]
	*Panax ginseng*	Ginsenosides (Rg1, Rb1)			[168]
Supporting cognitive function	*Glycyrrhiza uralensis*	Glycyrrhizin, Licorice polysaccharides	Enhance brain function, delay memory decline, improve cognitive ability		[130]
	*Lycium barbarum*	*Lycium barbarum* polysaccharides, flavonoids			[169]
	*Nelumbo nucifera*	*Nelumbo nucifera* polysaccharides, Flavonoids			[170]
	*Poria cocos*	*Poria cocos* polysaccharides, Triterpenoids			[131]
	*Panax ginseng*	Ginsenosides (RK1)			[171]

Abbreviations: FMH, Food-Medicine Homology; FSMP, Foods for Special Medical Purposes.

#### Others

In addition to the age groups discussed above, individuals with specific diseases, such as cancer, diabetes, cardiovascular disorders, rheumatic diseases, and gastrointestinal dysfunction require tailored nutritional support due to their unique physiological and metabolic demands.

Malnutrition and immunosuppression, common among patients with cancer, directly affect the tumor microenvironment and treatment response [64,[172], [173], [174]]. Therefore, incorporating anticancer plant bioactive compounds derived from *D. opposita* and *P. cocos* into FSMP can both inhibit tumor progression and improve nutritional status [99,175]. For metabolic diseases such as diabetes, nutritional interventions primarily focus on glycemic and metabolic regulation [176]. Diets rich in dietary fiber and antioxidants, including polysaccharides from *M. charantia* and *D. longan*, help improve insulin sensitivity and achieve blood glucose control [161,177,178,179]. Cardiovascular disease interventions extend to managing the circulatory system and oxidative stress [180,181], with polyphenols from *C. sinensis* and alkaloids from *Nelumbo nucifera* improving vascular function and exerting anti-inflammatory effects [182,183]. Notably, chronic inflammation is a key link connecting multiple diseases, especially evident in rheumatoid arthritis [184,185]. Joint symptoms can be alleviated by compounds such as curcumin and polysaccharides from *G. lucidum*, which possess clear anti-inflammatory and analgesic properties [186,187]. Moreover, all systemic nutritional support ultimately depends on efficient absorption and stable internal homeostasis, making gastrointestinal health critical. Supplementation with prebiotics, probiotics, and plant polysaccharides from *L. barbarum* and *Astragalus* can restore microbial balance and strengthen barrier function, thereby improving overall nutrient absorption [[188], [189], [190], [191], [192], [193]].

The use of FMH-derived natural products in FSMPs offers critical nutritional support and functional benefits for these patient populations. From cancer and diabetes to rheumatoid arthritis and gastrointestinal disorders, FMH-integrated FSMPs are increasingly positioned to meet the specialized needs of individuals with complex health conditions. As technological innovation and regulatory support advance, the medical food industry is expected to deliver more precise and personalized nutritional solutions, offering greater health benefits to those with special medical needs (Table 6) [99,101,120,127,152,160,162,175,179,178,182,183,186,187,[192], [193], [194], [195], [196], [197], [198], [199], [200], [201], [202], [203], [204], [205], [206], [207], [208], [209]].

**TABLE 6:** Overview of the application of FMH natural products in FSMPs for other special populations.

Target population	Demand characteristics	Sources	Main active ingredients	Application effects	Ref.
Patients with cardiovascular disease	Antioxidant and anti-inflammatory effects	*Lycium barbarum*	*Lycium barbarum* polysaccharides, flavonoids	Reduces oxidative stress, slows atherosclerosis, protects blood vessels from free radical damage, and prevents cardiovascular diseases	[194]
		*Morus alba*	Anthocyanins, flavonoids		[195]
	Improving blood pressure levels	*Curcuma longa*	Curcumin	Enhances blood flow, improves microcirculation, prevents thrombosis, and exerts anti-inflammatory effects	[196]
		*Camellia sinensis*	*Camellia sinensis* polyphenols, catechins		[182]
		*Nelumbo nucifera*	*Nelumbo nucifera* alkaloids, *Nelumbo nucifera* polysaccharides		[183]
		*Ziziphus jujuba*	*Ziziphus jujuba* polysaccharides, flavonoids		[197]
	Lowering blood lipids	Black *Lycium barbarum*	Black *Lycium barbarum* polysaccharides, flavonoids	Reduces blood lipids, improves cholesterol levels, prevents atherosclerosis, and supports cardiovascular health	[198]
		*Crataegus pinnatifida*	*Crataegus pinnatifida* flavonoids, triterpenes		[162]
		*Glycine max*	*Glycine max* isoflavones, saponins		[199]
Patients with cancer	Anticancer	*Camellia sinensis*	*Camellia sinensis* polyphenols, catechins	Reduces oxidative stress, slows aging, protects cells from free radical damage, and improves skin health	[200]
		*Morus alba*	Anthocyanins, flavonoids		[201]
	Enhancing immune function	*Astragalus*	*Astragalus* polysaccharides, *Astragalus* saponins	Enhances the immune system, inhibits tumor growth, reduces infections, and improves treatment tolerance	[152]
		*Ganoderma lucidum*	*Ganoderma lucidum* polysaccharides, triterpenes		[202]
	Improving digestion and absorption	*Dioscorea opposita*	*Dioscorea opposita* polysaccharides, diosgenin	Relieves indigestion, promotes appetite, and aids in the effective absorption of nutrients	[99]
		*Poria cocos*	*Poria cocos* polysaccharides, triterpenes		[175]
	Improving nutritional status	*Astragalus*	*Astragalus* polysaccharides, *Astragalus* saponins	Provides energy, alleviates fatigue, enhances immunity, and aids in physical recovery and strength	[203]
		*Panax ginseng*	Ginsenosides, saponins		[204]
		*Ziziphus jujuba*	Jujube polysaccharides, flavonoids		[205]
Patients with diabetes	Controlling blood sugar levels	*Astragalus*	*Astragalus* saponins, *Astragalus* polysaccharides	Lowers blood sugar, improves insulin sensitivity, controls postprandial blood glucose, and alleviates diabetes symptoms	[160]
		*Dimocarpus longan Dimocarpus longan*	*Dimocarpus longan* polysaccharides, flavonoids		[179]
		*Momordica charantia*	*Momordica charantia* saponins, *Momordica charantia* protein, polysaccharides		[178]
Patients with gastrointestinal dysfunction	Promoting digestion and absorption	*Dioscorea opposita*	*Dioscorea opposita* polysaccharides, diosgenin	Promotes gastrointestinal motility, enhances digestive absorption, improves appetite, and alleviates stomach discomfort	[99]
		*Poria cocos*	*Poria cocos* polysaccharides, triterpenes		[101]
		*Zingiber officinale*	Curcumin, gingerol		[127]
	Regulating gut microbiota and gut health	*Astragalus*	*Astragalus* polysaccharides, *Astragalus* saponins	Regulates gut microbiota, boosts immunity, alleviates constipation and diarrhea, and maintains gut health	[193]
		*Lycium barbarum*	*Lycium barbarum* polysaccharides, flavonoids		[192]
Patients with rheumatic disease	Anti-inflammatory effects	*Curcuma longa*	Curcumin	Anti-inflammatory, pain-relieving, reduces joint swelling, alleviates symptoms of rheumatoid arthritis, and reduces chronic inflammation	[186]
		*Ganoderma lucidum*	*Ganoderma lucidum* polysaccharides, triterpenes		[187]
		Moringa oleifera	Moringa oleifera polysaccharides, moringa oleifera saponins		[206]
	Enhancing immune function	*Astragalus*	*Astragalus* polysaccharides, *Astragalus* saponins	Boosts immunity, prevents rheumatic disease relapse, and helps reduce systemic inflammation	[120]
		*Ziziphus jujuba*	Jujube polysaccharides, flavonoids		[207]
	Improving joint function and repair	Fish oil	Omega-3 fatty acids (e.g., DHA, EPA)	Protects cartilage, reduces joint damage, enhances joint flexibility and comfort	[208]
		*Glycine max*	*Glycine max* isoflavones, saponins		[209]
		*Lycium barbarum*	Lycium barbarum polysaccharides, flavonoids		[160]

Abbreviations: FMH, Food-Medicine Homology; FSMP, Foods for Special Medical Purposes.

## Challenges and opportunities in the development of FSMPs from FMH natural products

### Challenges

The development of FMH-derived natural products for FSMP faces 3 major technical challenges: low extraction efficiency of active ingredients, complex purification processes, and poor storage stability. Overcoming these barriers is essential to realizing their full application potential, yet each remains a significant hurdle.

#### Extraction of active ingredients

**FIGURE 5:**
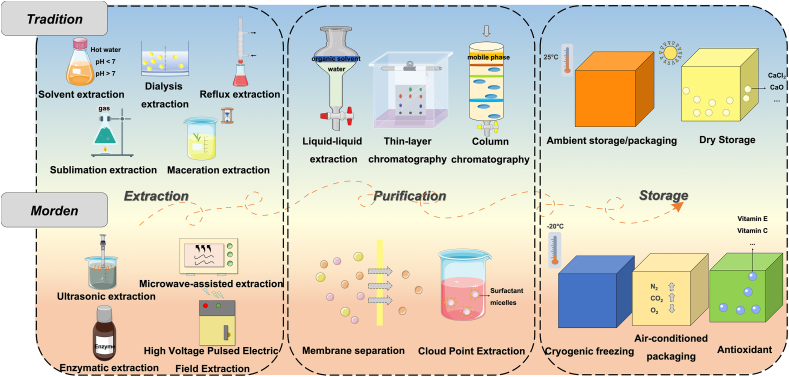
Optimization of Food-Medicine Homologous (FMH) bioactive compound processing. This figure summarizes key technological advances in the extraction, purification, and storage of FMH compounds, comparing conventional methods with modern techniques that improve efficiency and stability.

Efficient extraction of active ingredients is a critical step in developing FSMPs from FMH natural products, as it directly influences functional efficacy and overall application value. Traditional methods, such as hot water extraction, acid-base extraction, and distillation, are cost-effective and easy to perform. However, their high-temperature conditions often degrade thermolabile components like polysaccharides. This degradation can lead to glycosidic bond cleavage, conformational alterations, and subsequent loss of bioactivity [[210], [211], [212]]. In contrast, modern extraction techniques including ultrasound-assisted, microwave-assisted, and supercritical fluid extraction offer improved efficiency and greater environmental sustainability. Ultrasound facilitates extraction by disrupting cell structures, though excessive energy input may degrade sensitive compounds [213]. Microwave extraction, which employs electromagnetic radiation to rupture cell membranes, is both energy-efficient and environmentally friendly. However, high-intensity microwave exposure can also damage thermosensitive constituents [214,215]. Figure 5 presents representative traditional and modern extraction techniques.

FSMPs require higher levels of consistency, safety, and clinically validated efficacy. However, the structural complexity among FMH compounds poses significant challenges to standardization. Future research should prioritize integrated and mild extraction approaches such as ultrasound-microwave-assisted extraction and enzyme-assisted methods that maximize yield without compromising bioactivity. Equally important is upstream quality control, including standardized cultivation practices and contamination-free processing.

#### Purification of active ingredients

Purifying active compounds is a critical step after extraction, as it directly influences product purity, functional performance, and quality control. Traditional purification methods such as liquid–liquid extraction, column chromatography, and thin-layer chromatography are cost-effective and easy to implement. However, they often suffer from limitations, including low throughput, reduced yield, and high solvent consumption. For example, column chromatography, widely used in FMH purification, employs diverse stationary phases (e.g., C18, silica gel, macroporous resins) but often fails to resolve structurally similar compounds and can lead to loss of bioactivity [216,217]. In contrast, modern techniques like supercritical fluid extraction, membrane separation, and particularly cloud point extraction (CPE) offer higher efficiency and eco-friendliness. CPE separates hydrophilic and lipophilic compounds via surfactant-induced phase formation under controlled conditions, using minimal solvents and simple equipment, though its broader use is limited by sample and compound properties [218,219]. Figure 5 summarizes key traditional and modern purification techniques.

FSMPs demand not only high-purity ingredients but also preservation of functional integrity. Future approaches should move beyond single-step purification toward multistrategy integration, for example, combining membrane filtration with low-temperature supercritical fluid extraction to achieve gentle yet effective fractionation. Additionally, modular purification platforms with tunable parameters can improve adaptability across diverse FMH sources.

#### Storage stability of active ingredients

The storage stability of active ingredients extracted from FMH natural products directly influences the shelf life, safety, and bioactivity of FSMPs. Traditional storage methods often fail to protect heat-sensitive compounds and cannot fully prevent moisture and oxygen exposure. In contrast, modern technologies such as low-temperature freezing, vacuum packaging, inert gas protection, and antioxidant additives better preserve ingredient stability and slow oxidative degradation [220]. Modified atmosphere packaging, which creates low-oxygen, high-CO_2_ environments to inhibit oxidation and enzyme activity, is widely used in food preservation. However, it requires advanced equipment and incurs higher costs [220,221]. Figure 5 summarizes commonly used storage technologies.

Future storage strategies for FMH-based FSMPs should advance toward intelligent, precise systems that integrate real-time environmental monitoring, dynamic atmosphere control, and nano-encapsulation. These technologies can provide tailored protection for diverse, sensitive bioactives. Unlike traditional one-size-fits-all methods, adaptive storage maximizes functional preservation.

### Opportunities

The FSMP market is rapidly expanding, driven by 3 key factors: increasing chronic disease care needs, aging-related nutritional demands, and novel FMH applications amid the globalization of TCM. With proven safety and multifunctionality, FMH products are emerging as breakthrough innovations in FSMPs.

#### Demand and development trends in the FSMP market

The rapidly expanding FSMP market offers significant opportunities for integrating FMH bioactives. The market grew from $11.2 billion in 2019 to $13.5 billion in 2021 and is projected to reach $20 billion by 2030 [3]. Three key factors drive this growth. First, FSMPs are increasingly recognized for their clinical value in supporting malnutrition, cachexia, and cancer, often offering cost-effective alternatives to traditional treatments [3]. Second, the rising prevalence of chronic diseases like diabetes, projected to affect 783 million people, fuels demand for FSMPs tailored to nutritional support [222]. Third, the global population aged 60 and above is expected to surpass 2 billion by 2050 as declining metabolic and sensory functions heighten vulnerability to disease [223,224].

Currently, global FSMP demand remains concentrated in developed countries, with the United States representing 62% of the global share. In contrast, developing countries like China are emerging as key players in FSMP expansion due to their large populations and growing healthcare needs. In this context, FMH bioactive components, closely aligned with the principles of precision nutrition, are not merely auxiliary ingredients but represent strategic resources for driving future innovation and advancement in FSMP development.

#### Global spread of TCM

TCM has a history spanning over 3000 y and represents one of China’s invaluable intangible cultural heritages [[225], [226], [227]]. In 2019, the WHO included TCM in the 11th edition of the International Classification of Diseases, strengthening its role in global healthcare and laying a foundation for the worldwide application of FMH products [228]. In 2023, United States consumers spent $58 billion on supplements, with nearly half of cancer patients using such products [229]. The United States Food and Drug Administration (FDA) has approved 4 plant-derived drugs, supporting the integration of FMH into FSMPs, whereas the European Food Safety Authority has confirmed the safety of *Panax* notoginseng and *Astragalus* membranaceus extracts, now widely marketed globally [229,230]. Collectively, these trends highlight growing global acceptance of FMH products as FSMP ingredients.

However, realizing FMH’s full potential requires FSMP-specific research frameworks. We advocate leveraging TCM theory to identify promising FMH candidates, combined with targeted mechanistic and clinical studies, which can transform FMH from simple nutrients into functional agents that support immunity, metabolism, and gut health, bridging traditional wisdom and modern science.

#### Advantages of FMH natural products

FMH natural products hold significant promise for FSMP development due to their diverse health-promoting functions and high safety profile. They provide bioactive compounds with anti-inflammatory, antioxidant, antitumor, metabolic regulation, digestive support, and mood enhancement effects as discussed in Section 2.2. Moreover, FMH compounds show strong potential in addressing emerging diseases. For example, in post-COVID conditions, mogroside IIIE from *Siraitia grosvenorii* supports lung recovery by reducing inflammation and fibrosis [[231], [232], [233], [234]]. Safety is another key advantage. Curcumin holds Generally Recognized as Safe status from the United States FDA [235]. Additionally, 106 FMH substances listed by China’s Ministry of Health have undergone systematic toxicological evaluations, showing no significant adverse effects at standard dosages.

FMH natural products, with their proven efficacy, safety, and personalized nutritional benefits, are increasingly recognized as essential and forward-looking resources in FSMP development, addressing the evolving demands of health management.

In conclusion, this review systematically examines the potential applications, challenges, and opportunities of FMH bioactives in FSMP development. Within the FAM framework, FMH natural products provide essential nutrition and deliver anti-inflammatory, antioxidant, antitumor, hypoglycemic, hypolipidemic, and digestive-regulating effects. FMH-based FSMPs address the nutritional needs of diverse populations including infants, pregnant women, and the elderly offering a promising strategy for personalized nutrition and chronic disease management. This approach aligns with the global shift from pharmaceutical-centered interventions toward food-based, preventive care.

To fully translate FMH bioactives into effective FSMP products, several critical challenges require attention. These include optimizing bioactive extraction and stability, enhancing bioavailability and formulation compatibility, and navigating increasingly complex regulatory frameworks. Future research should focus on applying FMH principles effectively to FSMP development by leveraging multicomponent synergy while meeting modern demands. Concurrently, collaboration among regulators, industry, and healthcare sectors is essential to build an ecosystem that fosters FMH-based FSMP innovation through clear regulation, public engagement, and robust infrastructure support.

FMH-based interventions have the potential to reshape the FSMP landscape by offering culturally rooted, scientifically grounded, and clinically relevant nutritional healthcare solutions. As daily dietary interventions, they provide a unique opportunity for continuous, long-term health regulation and represent a vital element of future lifestyle-oriented medical nutrition strategies.

## Author contributions

The authors’ responsibilities were as follows – YZ, JL, YL, PA: conceived of and designed the project; XX, SH, XZ: drafted manuscript; YZ, JL, YL, SH, XX: revised the manuscript; and all authors: read and approved the final manuscript.

## Data availability

Data described in the manuscript, codebook, and analytic code will be made available on request pending application and approval.

## Funding

This work was supported by the National Key R&D Program of China (2022YFF1100402).

## Conflicts of interest

The authors report no conflicts of interest.
